# Path Planning of an Unmanned Aerial Vehicle Based on a Multi-Strategy Improved Pelican Optimization Algorithm

**DOI:** 10.3390/biomimetics9100647

**Published:** 2024-10-21

**Authors:** Shaoming Qiu, Jikun Dai, Dongsheng Zhao

**Affiliations:** 1 Key Laboratory of Network and Communications, Dalian University, Dalian 116622, China; daijikun@s.dlu.edu.cn; 2 School of Economics and Management, Ningxia University, Yinchuan 750021, China; z13103030546@163.com

**Keywords:** UAV, path planning, intelligent optimization algorithm, multi-objective optimization

## Abstract

The UAV path planning algorithm has many applications in urban environments, where an effective algorithm can enhance the efficiency of UAV tasks. The main concept of UAV path planning is to find the optimal flight path while avoiding collisions. This paper transforms the path planning problem into a multi-constraint optimization problem by considering three costs: path length, turning angle, and collision avoidance. A multi-strategy improved POA algorithm (IPOA) is proposed to address this. Specifically, by incorporating the iterative chaotic mapping method with refracted reverse learning strategy, nonlinear inertia weight factors, the Levy flight mechanism, and adaptive t-distribution variation, the convergence accuracy and speed of the POA algorithm are enhanced. In the CEC2022 test functions, IPOA outperformed other algorithms in 69.4% of cases. In the real map simulation experiment, compared to POA, the path length, turning angle, distance to obstacles, and flight time improved by 8.44%, 5.82%, 4.07%, and 9.36%, respectively. Similarly, compared to MPOA, the improvements were 4.09%, 0.76%, 1.85%, and 4.21%, respectively.

## 1. Introduction

**Figure 1:**
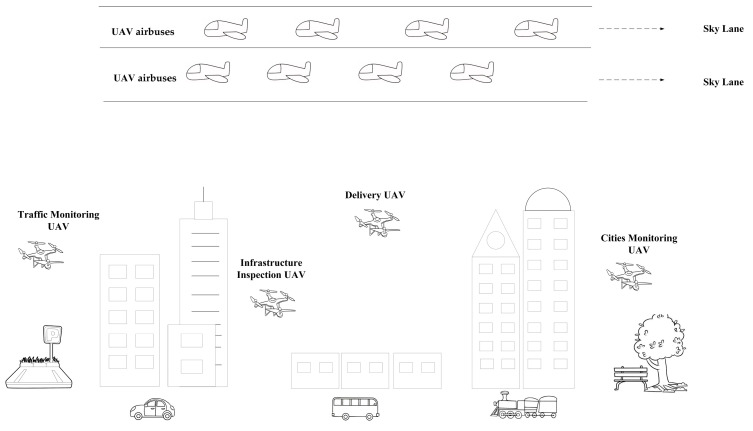
UAV flight scenarios. Mainly include traffic monitoring, express delivery, city inspection, etc.

With the rapid development of information technology, unmanned aerial vehicle (UAV) technology has attracted widespread attention across various application scenarios due to its flexibility and convenience [1]. UAVs are now extensively utilized in agriculture, disaster relief, transportation, and many other fields [2,3,4,5,6,7]. As UAV applications become more prevalent, the scenarios they encounter are increasingly complex and diverse. Figure 1 illustrates UAV flight scenarios, including traffic monitoring, express delivery, and urban inspection.

In urban environments, flight tasks such as delivery and inspection often prioritize finding shorter paths to improve efficiency. However, these flights inevitably encounter various obstacles, making effective trajectory planning essential. Currently, researchers are conducting extensive research on UAV path planning, utilizing traditional methods such as the Dijkstra algorithm [8], the A-star algorithm [9], and the rapidly expanding random tree (RRT) algorithm [10]. Due to the substantial computational time and resources required, this type of method is often difficult to apply in complex environments with significant terrain variations and diverse obstacle types. To address this multi-objective optimization problem, researchers have proposed intelligent optimization algorithms. Unlike traditional methods that rely on gradient information of the objective function, intelligent optimization algorithms search by directly computing the objective function values. They are not constrained by mathematical conditions such as continuous differentiability and can be easily applied to discrete optimization problems, such as integer programming [11]. The fundamental idea behind these algorithms is to simulate the collective behavior observed in nature and then quantify these characteristics to construct mathematical models applicable to a wide range of problems. Compared to traditional algorithms, intelligent optimization algorithms possess several advantages, including strong robustness, simplicity in computational logic, and powerful global search capabilities. Firstly, the individuals within the population of an intelligent optimization algorithm interact in a distributed manner, without a central control unit, ensuring that the failure of a few individuals does not compromise the overall problem-solving process, thus providing strong robustness. Secondly, by simulating collective behaviors found in nature, these algorithms effectively avoid being trapped in local optima, making it easier to find global optima. Additionally, each individual in the population perceives only local information and follows simple rules, resulting in a simple structure that is easy to implement.

The emergence of intelligent optimization algorithms has introduced new solutions to combinatorial optimization problems that traditional algorithms struggle to solve, and these algorithms have been successfully applied to optimize many complex engineering applications in practice [12,13,14]. Nevertheless, intelligent optimization algorithms still face several challenges in the context of UAV path planning. Firstly, although swarm intelligence optimization algorithms exhibit strong global search capabilities, they may still become trapped in local optima when dealing with complex multimodal problems, making it difficult to achieve global optima. Secondly, in certain cases, the convergence speed of intelligent optimization algorithms can be relatively slow, particularly when addressing complex problems, which may require a considerable amount of time to obtain a satisfactory solution. Additionally, although UAV task environments are primarily aerial, much of the research has focused on 2D scenarios. Even when 3D scenarios are considered, the simulations often involve simplistic obstacle models, lacking the detailed modeling and realistic flight simulations needed for more accurate representations of real-world environments.

The UAV path planning problem requires a comprehensive consideration of the algorithm, the map, and constraints. To address the aforementioned challenges, this paper proposes an improved Pelican Optimization Algorithm (IPOA). The main contributions of this paper are as follows:

(1). Based on the Pelican Optimization Algorithm (POA), a multi-strategy improved Pelican Optimization Algorithm (IPOA) is proposed. Specifically, by incorporating the iterative chaotic mapping method with refracted reverse learning strategy, nonlinear inertia weight factors, the Levy flight mechanism, and adaptive t-distribution variation, the convergence accuracy and speed of the POA algorithm are enhanced.
(2). Compared with the five intelligent optimization algorithms, the proposed algorithm reduces indicators such as the UAV flight path length, turning cost, iteration count, and distance from obstacles. The meanings of these indicators can be found in Table A2, Appendix A.

The rest of the paper is organized as follows: Section 2 introduces relevant research on intelligent optimization algorithms and UAV path planning. Section 3 describes the constraints of the UAV and our optimization goals. Section 4 presents our improved algorithm. In Section 5, five comparative algorithms are set up, and the performance of IPOA in different environments is validated from multiple perspectives using CEC2022 test functions and the Wilcoxon rank-sum test. In Section 6, complex three-dimensional terrains and emulated realistic terrain simulation experiments were conducted, and the results were analyzed. Finally, Section 7 summarizes the entire paper.

## 2. Related Studies

UAV path planning involves devising an optimal or satisfactory flight path from the initial point to the target point within a given flight area, taking into account flight constraints and the impact of algorithms. To ensure that the UAV safely reaches its destination, it is necessary to establish a practical mathematical model that considers factors such as flight distance, turning angles, and collision threats. In the context of complex maps and constrained mathematical models, developing an effective path planning algorithm remains a central focus of current research. This paper aims to advance the understanding of this field by focusing on two key research directions: traditional algorithms and intelligent optimization algorithms.

### 2.1. UAV Path Planning Based on Traditional Algorithms

Common traditional algorithms include the Dijkstra algorithm, A-star algorithm, rapidly exploring random tree (RRT), and Artificial Potential Field (APF). These algorithms are highly efficient in solving path planning problems in simple scenarios [15]. In Reference [16], a new Artificial Potential Field (D-APF) path planning algorithm was developed for UAVs following ground targets. This algorithm outperforms the conventional APF and is more suitable for UAV flying in environments with dynamic and unknown obstacles. In Reference [17], a method combining the Artificial Potential Field and an improved rapidly exploring random tree was introduced. This algorithm maintains computational efficiency while providing directional guidance for expanding nodes and has made progress in addressing issues such as slow convergence speed and unsmooth paths in UAV path planning. In Reference [18], a path planning algorithm based on obstacle Voronoi diagrams and the A-star algorithm was proposed for low-altitude UAV logistics scenarios. This algorithm successfully reduced the route length, flight time, and average network complexity while improving airspace coverage. However, when addressing path planning problems in complex environments, the large number of path nodes and the excessive computational load can expose the limitations of these algorithms. For example, while the A-star algorithm is relatively simple to implement, it has low search efficiency in large-scale, high-dimensional spaces and struggles to solve multi-constraint path planning problems [19]. Although RRT has a fast search speed, it is challenging to obtain an optimal flight path [20]. The APF algorithm, despite its fast planning speed and good real-time performance, can experience issues such as local oscillations and local minima in large-scale, high-dimensional spaces, which can render the flight path unusable [21]. Therefore, while traditional optimization algorithms can achieve good path planning results in simple environments, they are not efficient at solving autonomous UAV path planning problems in complex environments.

### 2.2. UAV Path Planning Based on Intelligent Optimization Algorithms

The optimization algorithm does not rely on the gradient information of the objective function but searches the solution space of the problem by defining a set of meta-heuristic operations and corresponding control strategies to find the optimal solution or a solution close to the optimal one. Therefore, in the face of complex multi-objective optimization problems, intelligent optimization algorithms can usually provide a higher-quality solution in a limited time. Common intelligent optimization algorithms include the genetic algorithm (GA) [22], particle swarm optimization (PSO) [23], ant colony optimization (ACO) [24], artificial bee colony (ABC) [25], gray wolf optimization (GWO) [26], and Harris Hawk Optimization (HHO) [27].

These algorithms have been applied to UAV path planning and have shown good results. Reference [28] proposed an algorithm that uses ACO, the Voronoi diagram, and clustering methods to enhance the GA initial population. This accelerates the convergence speed while significantly shortening the calculation time, providing an effective and feasible path for the UAV. Reference [29] improved the max–min ant colony algorithm using Cauchy mutants, enabling the algorithm to choose the shortest possible flight path for the UAV while avoiding collisions. Reference [30] proposed an improved artificial bee colony algorithm (IABC) based on multi-strategy synthesis, which allows UAV to quickly obtain the optimal path in complex environments. By comparing various traditional intelligent optimization algorithms, the feasibility and efficiency of the improved algorithm are verified. In Reference [31], the GWO algorithm was used to solve the trajectory planning problem of multiple UAVs in complex scenarios. The experimental results showed that the algorithm not only successfully plans a safe path but also minimizes the average flight time error between UAVs. To enhance the UAV performance in complex three-dimensional environments, Reference [32] introduced the Cauchy mutation strategy, adaptive weights, and the sine cosine algorithm (SCA) into the HHO algorithm, proposing an improved algorithm called SCHHO. Various simulation experiments showed that the proposed algorithm can generate more optimized UAV trajectory planning routes.

In recent years, several novel intelligent optimization algorithms have been proposed and successfully applied in many fields. These include the Beluga Optimization Algorithm (WOA) [33], which imitates the foraging behavior of beluga whales; the Sparrow Search Algorithm (SSA) [34], which imitates the foraging behavior of sparrows; the Sand Cat Swarm Optimization Algorithm (SCSO) [35], which imitates the living habits of sand cats; the Dung Beetle Optimization Algorithm (DBO) [36], which imitates the survival behavior of dung beetles; and the Subtractive Averaging Optimization Algorithm (SABO) [37], inspired by mathematical concepts. Inspired by pelican hunting, Pavel Trojovský and Mohammad Dehghani proposed the POA [38] in 2022, which has been successfully applied. However, POA still suffers from slow convergence and the inability to escape the local optima in path planning. A single deterministic intelligent optimization algorithm cannot adapt to all scenarios [39]. Therefore, to improve the efficiency of UAV path planning in complex environments, this paper proposes an improved Pelican Optimization Algorithm (IPOA) based on the POA.

## 3. Problem Model

UAV path planning is a constrained optimization problem, where the goal is to find a collision-free optimal path from the start point to the endpoint while satisfying all environmental and operational constraints. Due to the physical limitations of UAV power and flight, we typically have to consider optimization goals such as the UAV range cost, turning angle, and collision risk.

### 3.1. Three-Dimensional Terrain Model

During the flight of the UAV, the flight scene needs to be 3D modeled for path planning. The modeling formula can be expressed as:

(1) $$\begin{array}{l} z(x,y)=\sin(y+a)+b\cdot \sin(x)+c\cdot \cos(d\sqrt{y^{2}+x^{2}})+\\ e\cdot \cos(y)+f\cdot \sin(f\sqrt{y^{2}+x^{2}})+g\cdot \cos(y), \end{array}$$

where (x, y) corresponds to a point on the plane; z corresponds to the height of the point coordinates; and a, b, c, d, e, f, and g represent constant coefficients. By adjusting the constant coefficients, some characteristic landforms can be obtained.

To enrich the terrain, this paper overlaps the mountain model on this benchmark landform, as shown below:

(2) $$h(x,y)=\sum_{i}h_{i}e^{\left[-\frac{{\left(x-x_{i}^{c}\right)}^{2}}{a_{i}^{2}}-\frac{{\left(y-y_{i}^{c}\right)}^{2}}{b_{i}^{2}}\right]}+h_{o},$$

where $h_{0}$ is the base landform, $h_{i}$ is the altitude of the *i*-th peak, $\left(x_{i}^{c},y_{i}^{c}\right)$ is the central coordinate of the *i*-th peak, $a_{i}$ is the slope of the *i*-th peak on the *x*-axis, and $b_{i}$ is the slope of the *i*-th peak on the *y*-axis. Then, the final terrain model can be defined as:

(3) Z(x,y)=max[z(x,y),h(x,y)].

### 3.2. Flight Distance Cost

During the flight of a UAV, the length of the flight path will affect the UAV’s efficiency, so the flight distance cost is one of the important factors. Generally speaking, finding the shortest path can save a lot of time and resources. In this article, the flight distance is summarized by the Euclidean distance between each adjacent point, and its flight distance cost function can be expressed as:

(4) $$W_{1}=\sum_{i}\sqrt{{\left(x_{i+1}-x_{i}\right)}^{2}+{\left(y_{i+1}-y_{i}\right)}^{2}+{\left(z_{i+1}-z_{i}\right)}^{2}},$$

where $x_{i}$ is the *x*-direction coordinate value of the UAV at the *i*-th step, $y_{i}$ is the *y*-direction coordinate value of the UAV at the *i*-th step, and $z_{i}$ is the height value of the UAV at the *i*-th step.

### 3.3. Turning Angle Cost

**Figure 2:**
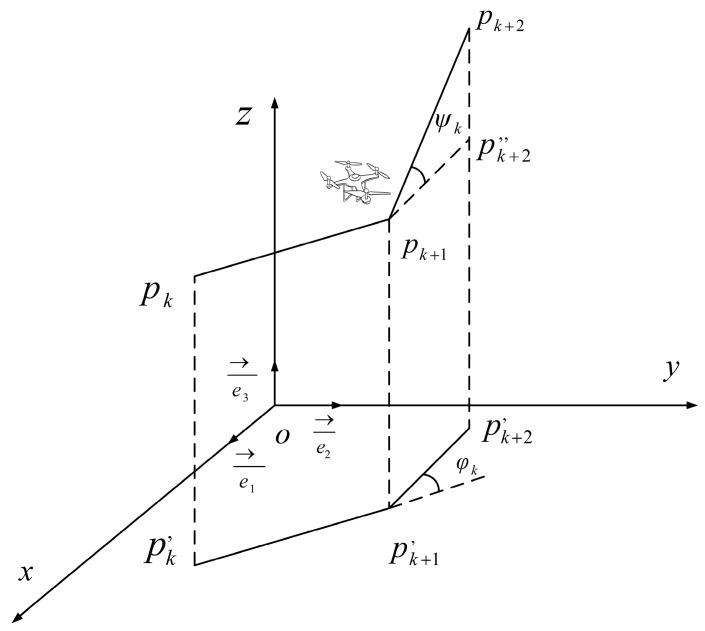
UAV flight 3D coordinate state during turns.

The UAV’s turning angle affects the smoothness and continuity of its path. Additionally, excessive turns can increase the energy consumption of the UAV. Therefore, the turning angle should be minimized during flight. The UAV can be regarded as a six-degree-of-freedom rigid body, and Figure 2 illustrates the UAV’s coordinate state during turns.

The turning angle $\varphi_{k}$ is the angle between two consecutive adjacent path segments. $\vec{e_{3}}$ is the unit vector in the direction of the *z*-axis, and the formula for calculating the projection vector is as follows:

(5) $$\vec{p_{k}^{\prime }p_{k+1}^{\prime }}=\vec{e_{3}}\times \left(\vec{p_{k}p_{k+1}}\times \vec{e_{3}}\right)$$

The formula for calculating the turning angle is:

(6) $$\varphi_{k}=\arctan\left(\frac{\left\|\vec{p_{k}^{\prime }p_{k+1}^{\prime }}\times \vec{p_{k+1}^{\prime }p_{k+2}^{\prime }}\right\|}{\vec{p_{k}^{\prime }p_{k+1}^{\prime }}\cdot \vec{p_{k+1}^{\prime }p_{k+2}^{\prime }}}\right)$$

where the notation · is a dot product, and × is a cross product.

The climbing angle $\psi_{k}$ is the angle between the $\vec{\left(p_{k}p_{k+1}\right)}$ and $\vec{\left(p_{k}^{,}p_{k+1}^{,}\right)}$, and the calculation formula is as follows:

(7) $$\psi_{k}=\arctan\left(\frac{z_{k+1}-z_{k}}{\left\|p_{k}^{\prime }p_{k+1}^{\prime }\right\|}\right)$$

The turning cost $W_{2}$ can be expressed as:

(8) $$W_{2}=a_{1}\cdot \sum_{k=1}^{n-1}\varphi_{k}+a_{2}\cdot \sum_{k=1}^{n-1}\left|\psi_{k}-\psi_{k-1}\right|$$

where $a_{1}$ and $a_{2}$ are constants.

### 3.4. Collision Threat Cost

During the flight, the UAV may encounter obstacles. To ensure the UAV’s safety, the relationship between the UAV and obstacles needs to be incorporated into the cost function. The definition of obstacle cost in this paper is as follows:

(9) $$\begin{array}{l} W_{3}=\left\{\begin{array}{ll} \lambda_{obs}\times \frac{1}{d_{obs}} & d_{obs}<d_{\mathrm{safe}\;}\\ 0 & d_{obs}>d_{\mathrm{safe}\;} \end{array}\right.\\ d_{obs}=\sum_{i=1}^{n}\sum_{j=1}^{S}\sqrt{{\left(x_{i}-x_{obs_{j}}\right)}^{2}+{\left(y_{i}-y_{osb_{j}}\right)}^{2}+{\left(z_{i}-z_{obs_{j}}\right)}^{2}}-\left(R_{uav}+R_{obs}\right) \end{array}$$

The variable $d_{obs}$ denotes the distance between the current node and an obstacle, while n is the number of nodes, and S is the number of obstacles. The coordinates of the current node are expressed as ($x_{i},y_{i},z_{i}$), and the coordinates of a specific obstacle are given as $\left(x_{{obs}_{j},}y_{{obs}_{j}},z_{{obs}_{j}}\right)$. Additionally, R corresponds to the radius of the UAV, $R_{obs}$ to the radius of the obstacle, $d_{safe}$ to the safety distance, and $\lambda_{obs}$ to the penalty coefficient for the obstacles.

### 3.5. Optimize the Target

In the whole planning process, it is necessary to comprehensively consider the shortest track length, the smallest possible flight angle, and the smallest track threat. Therefore, the overall cost function of path planning can be defined as:

(10) $$f\left(\omega_{i},W_{i}\right)=\sum_{i=1}^{3}\omega_{i}W_{i},$$

where $\omega_{i}$ is the weight parameter, and $\sum_{i=1}^{3}\omega_{i}=1$.

### 3.6. Path Smoothing

The path planning algorithm generates many discrete points, which cannot directly meet the flight requirements of the UAV. Therefore, these discrete and unsmooth sample points must be converted into a smooth path. In this paper, a cubic B-spline [40] interpolation algorithm is used to generate a smooth flight path:

(11) $$S(u)=\sum_{i=0}^{n}P_{i}\;\cdot \;N_{i,p}\left(u\right),0\leq u\leq u_{\max}$$

where $P_{i}$ is the control point, and $N_{i,p}(u)$ is the basis function of the B-spline. The basis function $N_{i,p}(u)$ can be written as:

(12) $$\begin{array}{c} N_{i,0}(u)=\left\{\begin{array}{l} 1,u_{i}\leq u\leq u_{i+1}\\ 0,others\; \end{array}\right.\\ N_{i,k}(u)=\frac{u-u_{i}}{u_{i+k}-u_{i}}\cdot N_{i,k-1}(u)+\frac{u_{i+k+1}-u}{u_{i+k+1}-u_{i+1}}\cdot N_{i+1,k-1}(u) \end{array}$$

## 4. Improved Pelican Optimization Algorithm (IPOA)

### 4.1. Pelican Optimization Algorithm

#### 4.1.1. Population Initialization

POA [38] operates as a population-based algorithm, with pelicans serving as its individuals. In swarm-based bio-inspired algorithms, each member of the swarm represents a candidate solution. These members propose values for optimization variables based on their positions in the search space. Initially, the population is randomly initialized within the problem’s defined lower and upper bounds, expressed as:

(13) $$x_{i,j}=l_{j}+rand\cdot (u_{j}-l_{j}),i,j=1,\;2,\ldots ,m$$

where $x_{i,j}$ represents the value of the j-th variable of the i-th candidate solution, N denotes the number of population members, m is the dimensionality of the problem variables, rand denotes a random number in the interval [0, 1], and $l_{j}$ and $u_{j}$ are the lower and upper bounds of the j-th problem variable, respectively.

Following this, the pelicans begin their hunting phase, which can be divided into two stages: pelicans moving towards prey (exploration phase) and pelicans gliding over the water surface (exploitation phase).

#### 4.1.2. Exploration Phase

For each pelican, its new position is updated based on its current position and the position of its prey. If the objective function value at the new position is less than that of the prey, the pelican moves towards the prey; otherwise, it moves away from the prey. The position update formula is given by:

(14) $$x_{i,j}^{P_{1}}=\left\{\begin{array}{l} x_{i,j}+\mathrm{rand}\cdot \left(p_{j}-I\cdot x_{i,j}\right),\;F_{p}<F_{i}\\ x_{i,j}+\mathrm{rand}\cdot \left(x_{i,j}-p_{j}\right),\;F_{p}⩾F_{i} \end{array}\right.$$

where $x_{i,j}^{P_{1}}$ represents the new state of the i-th pelican in the j-th dimension during the exploration phase. $p_{j}$ denotes the position of the prey in the j-th dimension, and $F_{p}$ is its objective function value. The parameter I is a random integer that can be either 1 or 2. This parameter is randomly chosen for each iteration and each member. When I = 2, it increases the displacement of each individual, enabling them to explore new regions in the search space.

If the objective function value at the new position is less than that at the original position, then the new position is accepted; otherwise, the original position remains unchanged. This acceptance criterion can be expressed as:

(15) $$x_{i}=\left\{\begin{array}{ll} x_{i}^{P_{1}}, & F_{i}^{P_{1}}<F_{i}\\ x_{i}, & F_{i}^{P_{1}}⩾F_{i} \end{array}\right.$$

#### 4.1.3. Exploitation Phase

After reaching the water surface, pelicans spread their wings and move upwards towards the fish before gathering them into their throat pouches. This strategy leads to capturing more fish in the targeted area. For each pelican, a new position is randomly generated nearby, and its objective function value is computed. The formula for updating the new position is:

(16) $$x_{i,j}^{P_{2}}=x_{i,j}+R\cdot \left(1-\frac{t}{T}\right)\cdot (2\;\cdot \;\mathrm{rand}\;-1)\cdot x_{i,j}$$

where $x_{i,j}^{p_{2}}$ represents the new state of the i-th pelican in the j-th dimension during the exploitation phase. Here, t is the iteration counter, and T is the maximum number of iterations. The term ($1-\frac{t}{T}$) denotes the neighborhood radius of $x_{i,j}$, allowing for local searches around each member to converge towards better solutions within the population. R is a parameter that can be set between 0 and 1, and rand is a random number between 0 and 1.

If the objective function value at the new position is less than that at the original position, then the new position is accepted; otherwise, the original position remains unchanged. This can be expressed as:

(17) $$x_{i}=\left\{\begin{array}{ll} x_{i}^{P_{2}}, & F_{i}^{P_{2}}<F_{i}\\ x_{i}, & F_{i}^{P_{2}}⩾F_{i} \end{array}\right.$$

### 4.2. Multi-Strategy Improved Pelican Optimization Algorithm (IPOA)

#### 4.2.1. Iterative Chaotic Mapping and Refracted Opposition-Based Learning

**Figure 3:**
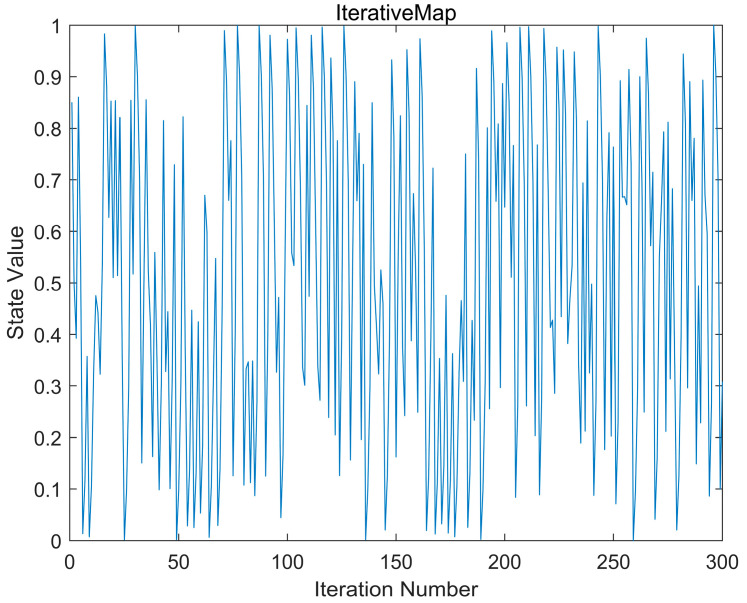
Iterative chaotic mapping.

Chaotic mapping is a type of nonlinear dynamic behavior. The unpredictability and aperiodicity of chaos can effectively enhance the optimization efficiency of algorithms. The basic idea is to linearly map the optimization variables to chaotic variables through chaotic mapping, then conduct optimization searches based on the ergodicity and randomness of chaos and, finally, linearly transform the obtained solutions back to the optimization variable space [41]. The definition of iterative chaotic mapping is as follows:

(18) $$z_{k+1}=\sin(\frac{a\pi }{z_{k}})$$

where a∈(0,1) is the control parameter. When a is set to 0.7 and the Iterative mapping is run for 300 iterations, the results are shown in Figure 3.

Opposition-Based Learning (OBL) [42], proposed by Tizhoosh H R, is an optimization strategy aimed at expanding the search range of the population. The core idea is to compute the opposite solution of the current solution and then select the better solution for iteration to find a more optimal solution for the given problem, thus improving the optimization performance of the algorithm. However, in later stages, the opposite solution may fall into a local optimum region.

**Figure 4:**
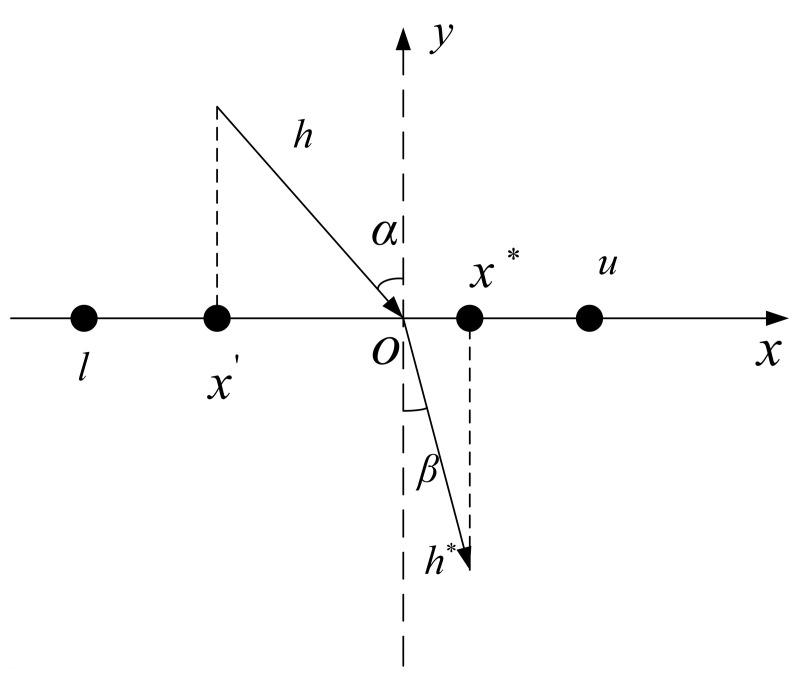
Refracted Opposition-Based Learning.

To address this issue, Refracted Opposition-Based Learning (ROBL), which combines the principles of light refraction, was proposed. ROBL can improve the algorithm performance and expand the search space to various degrees. The principle is illustrated in Figure 4.

In Figure 4, the *x*-axis represents the search optimization range [l, u], and the *y*-axis represents the normal line. α and β represent the incident and refraction angles, respectively, while h and $h^{*}$ represent the lengths of the incident and refracted rays. O is the midpoint of the search optimization range.

According to the definition of the refractive index, we can obtain:

(19) $$n=\frac{h^{*}((l+u)/2-x^{\prime })}{h\left(x^{*}-(l+u)/2\right)}$$

Let the scaling factor k = h/$h^{*}$ and substitute it into Formula (19) to obtain:

(20) $$x^{*}=\frac{l+u}{2}+\frac{l+u}{2kn}-\frac{x^{\prime }}{kn}$$

In this paper, the initial pelican population is generated using an iterative chaotic mapping method combined with the ROBL. This approach leverages the characteristics of the strategy to ensure a uniform distribution of search agents, allowing for a more comprehensive exploration of the search space within a certain range, making it easier to escape the local optima and enhancing the convergence speed. The mathematical formula is defined as:

(21) $$\left\{\begin{array}{c} x_{i,j}=chaotic\_map\left(x_{init},a\right)\\ x_{i,j}^{⋆}=\frac{l_{j}+u_{j}}{2}+\frac{l_{j}+u_{j}}{2kn}-\frac{x_{i,j}}{kn} \end{array}\right.$$

where $x_{init}$ is the initial population.

The specific steps are as follows:

(1). Randomly generate N candidate positions using iterative chaotic mapping to construct population $S_{1}$.
(2). Apply the ROBL to determine the refracted opposition population $S_{2}$ from population $S_{1}$.
(3). Integrate populations $S_{1}$ and $S_{2}$. Sort the combined population in descending order based on individual fitness values and select the top N pelican individuals with the highest fitness values to form the initial pelican population.

#### 4.2.2. Nonlinear Inertia Weight Factor

Due to the close correlation between the update of pelican individual positions and their current locations, to better balance the global exploration capability and local exploitation capability of the POA and to enhance the convergence accuracy, this paper employs a nonlinear inertia weight factor ω to adjust the relevance between pelican position updates and their current positions. The calculation method of the nonlinear inertia weight factor is shown:

(22) $$\omega =\exp(-\frac{t}{T})$$

In the initial iterations of the algorithm, when ω is small, the update of individual positions is minimally affected by the current pelican’s position. This favors the algorithm to explore a larger range, thereby enhancing its global exploration capability. As the optimization progresses, ω gradually increases, amplifying the influence of the current pelican’s position on individual position updates. At this stage, the algorithm narrows its optimization scope, aiding in the search for the optimal solution. This strategy not only enhances the algorithm’s capability for local exploration but also accelerates its convergence speed. The improved pelican calculation formula is as follows:

(23) $$x_{i,j}^{P_{1}}=\left\{\begin{array}{l} \omega \cdot x_{i,j}+\mathrm{rand}\cdot \left(p_{j}-I\cdot x_{i,j}\right),\;F_{p}<F_{i}\\ \omega \cdot x_{i,j}+\mathrm{rand}\cdot \left(x_{i,j}-p_{j}\right),\;F_{p}⩾F_{i} \end{array}\right.$$

#### 4.2.3. Levy Flight

As pelicans fly towards nearby prey, with increasing iterations, they are prone to getting trapped in local optima near the optimal solution. To address this issue, this study introduces a Levy flight during the developmental stage of pelicans, mutating individual pelicans to expand their search radius, enhance population diversity, avoid local optima, and strengthen global exploration capabilities.

The Levy flight is a random walk strategy applicable to natural behaviors such as group foraging. Here, the variable Levy(λ) follows a Levy distribution with parameter λ, expressed as:

(24) $$Levy(\lambda )\sim u=t^{-\lambda },1<\lambda <3$$

where λ represents the exponent, which significantly complicates the computation. Therefore, the Mantegna algorithm is employed to compute the Levy flight path:

(25) $$s=\frac{u}{{\left|v\right|}^{\frac{1}{\beta }}}$$

where *s* denotes the Levy flight path factor, and β is a parameter controlling the step size, with 0 < β < 2. To balance global and local search capabilities, β is set to 1.5. *u* and *v* are random values from a Gaussian distribution, with $u\sim N\left(0,\sigma_{u}^{2}\right)$ and $v\sim N\left(0,\sigma_{v}^{2}\right)$. The definitions of $\sigma_{u}$ and $\sigma_{v}$ are as follows:

(26) $$\left\{\begin{array}{c} \sigma_{u}={\left\{\frac{\Gamma (1+\beta )\sin\left(\frac{\beta \pi }{2}\right)}{\Gamma \left(\frac{1+\beta }{2}\right)\beta \cdot 2\frac{\beta -1}{2}}\right\}}^{\frac{1}{\beta }}\\ \sigma_{v}=1 \end{array}\right.$$

The improved calculation formula is as follows:

(27) $$x_{i,j}^{P_{2}+1}=s\cdot x_{i,j}^{P_{2}}+x_{i,j}^{P_{2}}$$

#### 4.2.4. Adaptive t-Distribution Variation

T-distribution variation is an information perturbation strategy, which is defined as follows:

(28) $$x_{i}^{*}=x_{i}+x_{i}\cdot t(v)$$

where $x_{i}$ represents the current position of the individual, while $x_{i}^{*}$ represents the position of the individual after mutation. t(v) is a random variable drawn from a t-distribution with degrees of freedom v.

The parameter v dynamically adjusts with the number of iterations to balance global exploration and local exploitation and is defined as:

(29) $$\nu =\nu_{\min}+\left(\nu_{\max}-\nu_{\min}\right)\times \frac{t}{T}$$

where $v_{min}$ is the minimum degree of freedom at the beginning, $v_{max}$ is the maximum degree of freedom at the end, t is the current iteration number, and T is the maximum number of iterations.

**Figure 5:**
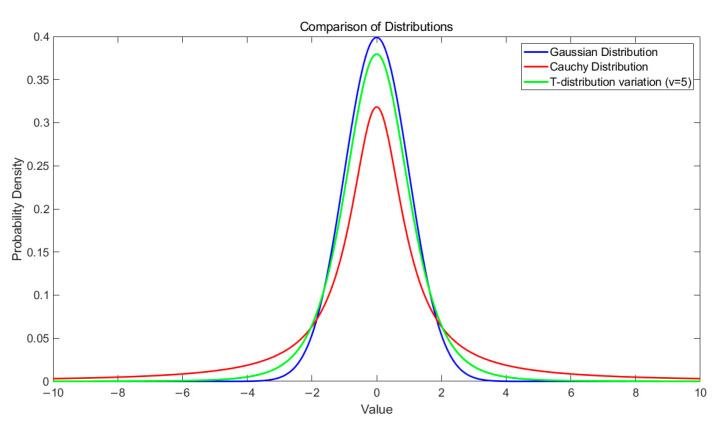
The density distribution of the three functions.

The degree of freedom affects the shape of the curve: as v approaches infinity, the curve appears a Gaussian distribution N(0, 1); as v approaches 1, the curve appears a Cauchy distribution C(0, 1). The density functions of these three distributions are illustrated in Figure 5.

In the early stages of the algorithm’s execution, the degree of freedom is set to a smaller value due to the lower number of iterations, resulting in a flatter curve in the middle. At this point, the algorithm exhibits strong global exploration capabilities. As the number of iterations increases, v gradually increases, causing the middle of the curve to become more pronounced. At this stage, the algorithm demonstrates enhanced local exploitation abilities. Therefore, the t-distribution variation effectively combines the characteristics and advantages of both the Cauchy and Gaussian distributions, offering robust global exploration and local exploitation capabilities.

The adaptive t-distribution variation does not alter the original algorithm’s update principle. Therefore, the improved position formula is defined as follows:

(30) $$x_{i,j}^{t}=x_{best}+x_{best}\cdot t\left(v\right)$$

where $x_{i,j}^{t}$ denotes the mutated state of the pelican individual, and $x_{best}$ represents the current best position.

### 4.3. The Detailed Process of IPOA

At the beginning of the algorithm, the population is initialized using a strategy that combines chaotic mapping with Refraction Opposition-Based Learning (ROBL). Next, the fitness values of each individual are calculated, and the position representing the optimal solution, referred to as the prey position, is generated to guide the behavior updates of the pelican population. During the exploration phase, the adjustment of nonlinear inertia weights dynamically influences the pelicans’ search behavior, directly affecting the process of updating individual positions. In the exploitation phase, the Levy flight mechanism is introduced to extend the search radius, allowing pelicans to explore areas further from the prey, enhancing the algorithm’s global search capability. Finally, the adaptive t-distribution variation is applied to fine-tune the current positions of pelican individuals, generating new candidate solutions and exploring new boundary regions. These mutated individuals are then subjected to fitness recalculation, forming a closed iterative loop.

**Figure 6:**
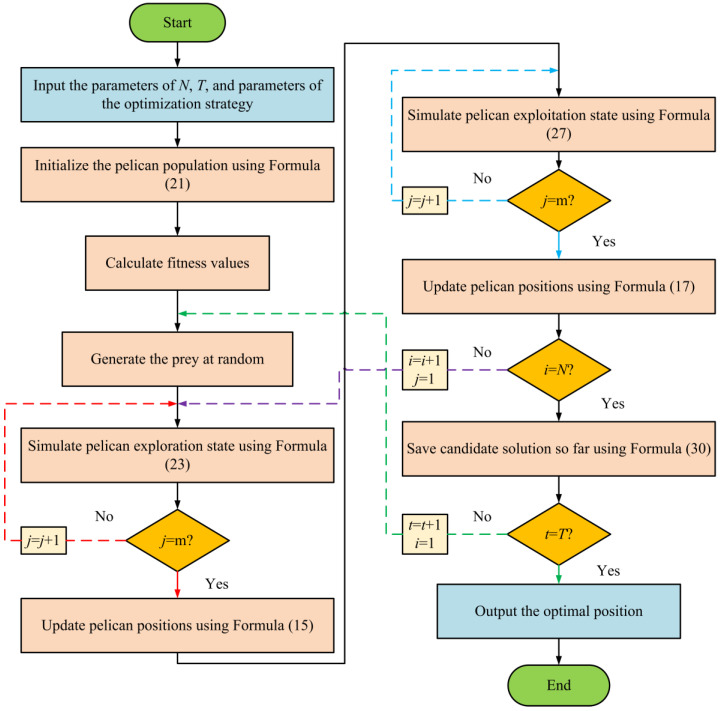
IPOA flowchart.

The steps of the proposed IPOA are presented in the form of a flowchart in Figure 6 and as pseudocode in Algorithm 1.
**Algorithm 1:** Pseudo-code of IPOA**Input:** Maximum number of iterations *T*, population size *N*, Improve policy parameters:   Chaotic mapping constant *a*, Nonlinear inertia weight factor *ω*, Levy mechanism   constant *β*, degree of freedom *v*, etc.**Output:** The best location *xbest*.**1**  Input various parameters;**2**  Use Formula (21) to initialize the population;**3**  Calculating the fitness of the individual by the objective function;**4  for** t = 1:*T* **do****5**Update the weight factor ω according to Formula (22), Update the degree of freedom *v* according to Formula (29);**6**Generate the position of the prey at random.;**7****for** i = 1:*N* **do****8**
Phase 1: Moving towards prey (exploration phase);**9**
**for** j = 1:*m* **do****10**

Calculate new status of the j-th dimension using Formula (23);**11**
**end****12**
Update the i-th population member using Formula (15);**13**
Phase 2: Winging on the water surface (exploitation phase);**14**
**for** j = 1:*m* **do****15**

Calculate new status of the j-th dimension using Formula (27);**16**
**end****17**
Update the i-th population member using Formula (17);**18****end****19**Get the current new location using Formula (30);**20  end****21**  Output best candidate solution obtained.

POA has a time complexity of O(*N* × *D* × *T*), where *N* is the population size, *D* is the dimensionality, and *T* is the maximum number of iterations. IPOA incorporates four additional components. Iterative chaotic mapping involves a one-dimensional nonlinear mapping with a time complexity of O(*N* × *D*). ROBL is designed to select pelican individuals, so integrating this strategy with iterative chaotic mapping does not alter the original algorithm’s complexity. The introduction of a nonlinear inertia weight factor, the Levy flight mechanism, and adaptive t-distribution variation are intended to modify the position update mechanism, which does not change the original algorithm’s time complexity. Therefore, IPOA’s time complexity remains O(*N* × *D* × *T*) + O(*N* × *D*) = O(*N* × *D* × *T*), which is comparable to POA in terms of time complexity.

## 5. Algorithm Comparison Experiments

To ensure objectivity, all experiments were conducted on a 64-bit Windows 11 operating system, utilizing an i7-11800H CPU and an RTX 3060 GPU. Matlab R2017b served as the simulation software for the experiments.

### 5.1. Experimental Design

To assess the performance of the algorithms, this paper chose five as baselines: DBO [36], HHO [27], SSA [34], POA [38], and an improved algorithm, MPOA, which has been applied in reservoir porosity prediction [43]. These algorithms are all excellent intelligent optimization algorithms and have been widely applied in many fields. They were selected because each has different characteristics. HHO is an easy-to-implement and cost-effective algorithm, SSA is widely used in the field of path planning, DBO is a recent research achievement, POA is the prototype algorithm for IPOA, and MPOA represents another improvement approach from other literature. Choosing these algorithms as baseline models ensures that they can fairly reflect the advantages of the new algorithm while ensuring the rationality of the experimental design and the interpretability of the results.

These algorithms were evaluated using the CEC2022 test functions, detailed in Table 1. Table 2 summarizes the key parameter settings for each algorithm, with the meanings of the parameters referenced in Table A1, Appendix A. Each test function was executed independently 30 times, with a maximum of 1000 iterations, a population size of 30, and a dimensionality of 20.

### 5.2. Comparative Analysis of Algorithms

The performance of the algorithms was evaluated using the best value, mean, and standard deviation (SD). The best value refers to the result found by the algorithm that is closest to the global optimum across multiple runs. It reflects the best performance of the algorithm in solving a specific problem. The mean value refers to the average of all results obtained by the algorithm over multiple runs. It provides an overall view of the algorithm’s performance across multiple experiments. The standard deviation refers to the degree of dispersion of the results of the algorithm over multiple runs. Smaller best values and means indicate higher convergence accuracy, while smaller standard deviations suggest more stable optimization. Since these three metrics can evaluate and compare the performance and stability of different optimization algorithms, we selected them as evaluation criteria.

**Figure 7:**
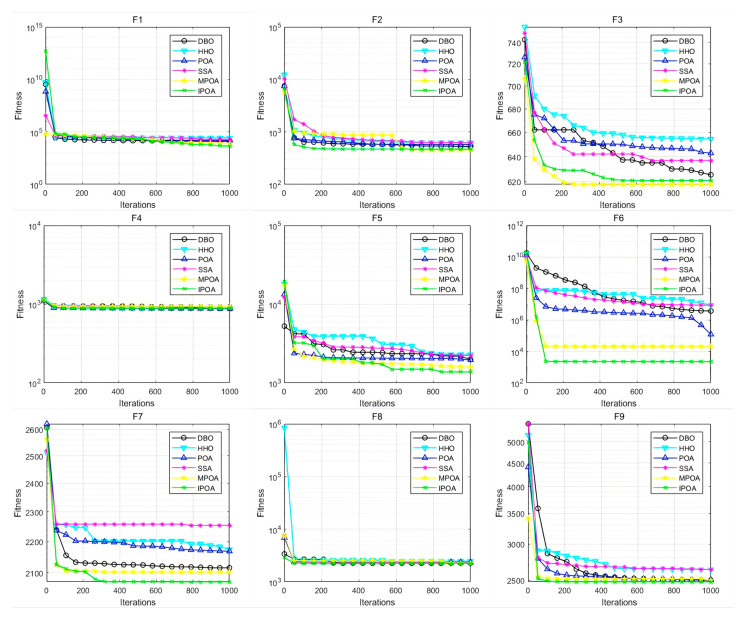

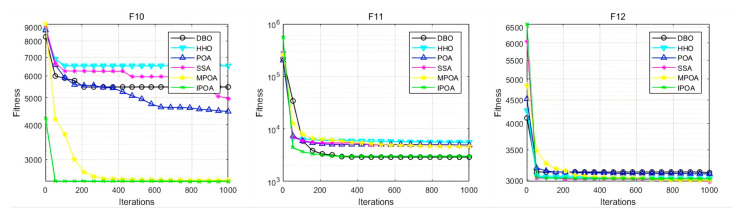
Convergence curves of the algorithms.

The numerical performance of the algorithms and their rankings are presented in Table 3. To provide a more intuitive observation and comparison of the algorithms’ convergence behavior, convergence curves for the six algorithms are plotted, as shown in Figure 7.

From Table 3 and Figure 7, it can be seen that IPOA achieves higher solution accuracy on the unimodal function $f_{1}$, indicating that IPOA’s local search capability is significantly stronger than the comparison algorithms. Similarly, IPOA demonstrates the best performance and stability on the multimodal function $f_{5}$ and also leads on $f_{2}$, $f_{3}$, and $f_{4}$, and although some metrics slightly lag behind MPOA, the numerical difference is minimal. In the hybrid functions, IPOA shows outstanding performance and stability on functions $f_{6}$ and $f_{7}$ while also leading on $f_{8}$, with only a minor difference from the best result. In the composite functions, IPOA achieves the best optimization results and stability on functions $f_{10}$ and $f_{12}$ while also performing excellently on $f_{9}$ and $f_{11}$. Overall, across 36 metrics in three categories, IPOA reaches the optimal performance in 25 metrics, proving its strong capabilities in various engineering applications.

**Figure 8:**
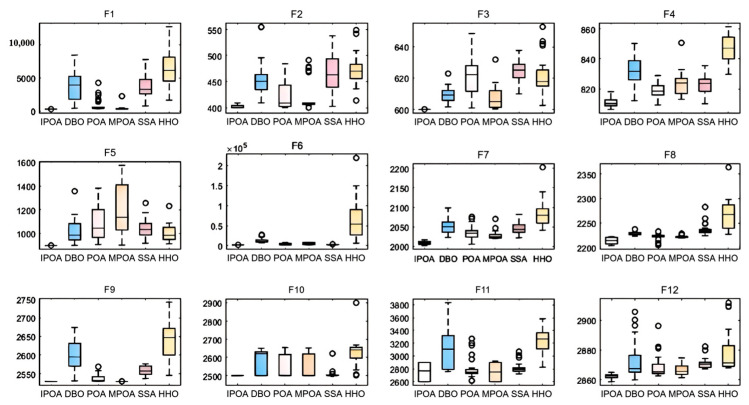
Box plots of the comparative algorithms.

To observe the performance of IPOA from more perspectives, we adjusted the dimensionality to 10, resulting in the box plots shown in Figure 8.

It can be observed that, in $f_{1}$, $f_{3}$, $f_{4}$, $f_{5}$, $f_{7}$, and $f_{12}$, IPOA has an obvious minimum value. In the other functions, IPOA either has an indistinct minimum value or is close to the minimum. In $f_{2}$, $f_{3}$, $f_{4}$, $f_{5}$, $f_{7}$, $f_{8}$, and $f_{12}$, IPOA’s median value is also significantly the lowest. In the remaining functions, IPOA shows either an indistinct lowest median or one close to the minimum. Almost all the medians are located near the center of the box, indicating that the data distribution is roughly symmetrical. Except for $f_{8}$, IPOA has the smallest box length in all the functions, indicating strong stability. From this analysis, it is evident that IPOA demonstrates high performance and robustness across various test environments.

To comprehensively assess the performance of the algorithm, the Wilcoxon rank-sum test was introduced for independent comparisons to verify if there are significant differences between IPOA and the other algorithms. The null hypothesis was set as there is no significant difference between the two algorithms being compared. If the significance level is less than 0.05, the null hypothesis is rejected, indicating a significant difference between the two algorithms and superior optimization by IPOA. If the result is greater than 0.05, the null hypothesis is not rejected, indicating no significant difference between the two algorithms, and their optimization effects are limited. The results of comparing IPOA with the other five algorithms are presented in Table 4.

From the data in Table 4, it can be seen that most of the significance level results are less than 0.05, indicating that IPOA is successful in most cases. However, in functions $f_{2}$, $f_{8}$, and $f_{11}$, some algorithms have significance levels greater than 0.05. Additionally, in functions $f_{5}$, and $f_{10}$, many algorithms show significance levels mostly greater than 0.05. This suggests that no single algorithm excels in all scenarios. Therefore, for UAV path planning, further experiments are needed to validate the algorithm’s performance.

## 6. UAV Path Planning Experiment

### 6.1. Simulation Experiment Setup

To evaluate the IPOA’s path planning capability in a complex 3D environment, simulated flight experiments were conducted. The experimental setup was configured as described in Section 5. The map size was [100, 100, 350]. At the same time, we used DBO, POA, HHO, SSA, and MPOA as comparison algorithms. For fairness, the population size for all algorithms was set to 30, with a maximum of 1000 iterations, and each algorithm was run independently 10 times. The best value, mean value, and standard deviation were used as statistical metrics to assess the optimization performance.

### 6.2. Effect of the Cost Function Parameters

In path planning, each path is encoded as a set of vectors composed of cost functions. The lowest total cost value, calculated by the intelligent optimization algorithm, identifies the corresponding path. Therefore, the weight allocation of the components in the cost function has a significant impact on the path. In this section, three different weight combinations, labeled as {$\omega_{1}=0.7,\omega_{2}=0.15$, $\omega_{3}=0.15$}, {$\omega_{1}=0.15,\omega_{2}=0.7$, $\omega_{3}=0.15$}, and {$\omega_{1}=0.15,\omega_{2}=0.15$, $\omega_{3}=0.7$}, representing a focus on the path length, turning, and collision avoidance, respectively, explore the algorithm’s performance under these different weight configurations. The performance of the various functions is shown in Table 5.

It can be observed that IPOA achieved optimal results in seven out of nine metrics. In Experiment 1, IPOA reached the best performance across all three metrics among all algorithms. In Experiment 2, IPOA achieved the best results in both the optimal value and mean value, with the standard deviation being slightly behind MPOA but with a minimal difference. In Experiment 3, IPOA achieved the best mean value and standard deviation, and although the optimal value was second to MPOA, the difference was minimal. Overall, IPOA demonstrated excellent searchability and robustness across all test scenarios.

### 6.3. Random Map Experiment

In real-world situations, UAVs inevitably have to make turns during flight. Additionally, to absolutely prevent collisions, a sufficiently large safety distance is typically set. In practical missions, UAVs focus more on task efficiency. We believe that the weighting of turning costs and collision costs should be lower than the path cost. Therefore, we opted to assign the weights as {$\omega_{1}$ = 0.7, $\omega_{2}$ = 0.15, $\omega_{3}$ = 0.15} for our experiments.

**Figure 9:**
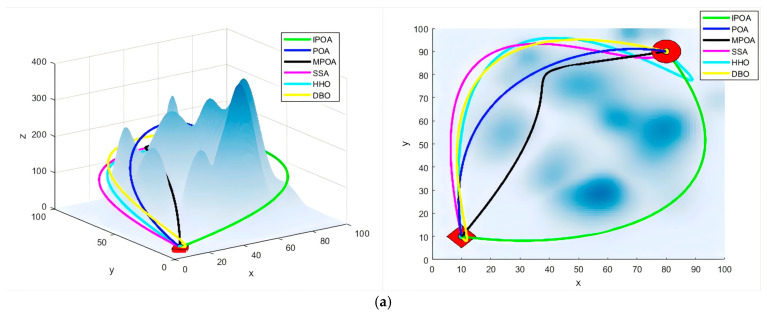

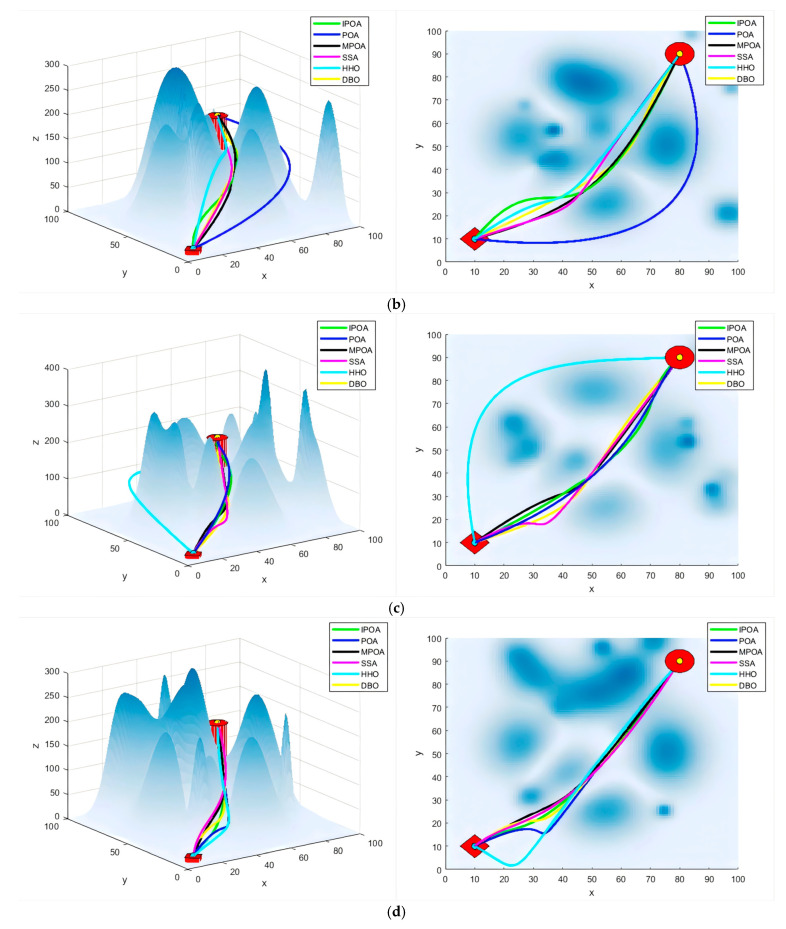

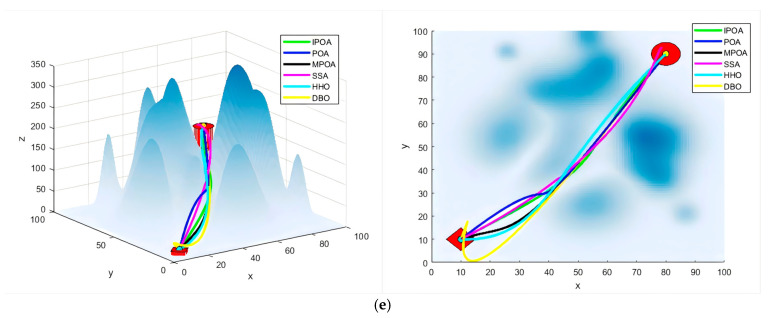
Flight simulation experiment of random maps (obstacles = 8). (**a**) Experiment 1; (**b**) Experiment 2; (**c**) Experiment 3; (**d**) Experiment 4; (**e**) Experiment 5.

We set the number of obstacles to eight and randomly generated five different sets of maps for the experiments. To more clearly demonstrate the flight results, we presented both the front view (left) and top view (right) of the flight paths for each algorithm in Figure 9. Table 6 records the evaluation metrics, where “Distance” represents the average path length, “Iterations” indicates the average number of iterations of the algorithm, “Turn” denotes the average deflection angle difference between two adjacent nodes on the path, and “Dis-obs” represents the average distance between the current node and the nearest obstacle among the nodes on the path.

In Experiment 1, IPOA achieved the optimal average flight distance and the minimum average distance from obstacles. Its average convergence rate was second only to MPOA, with only a slight difference between the two. However, IPOA performed poorly in terms of the average turning angle. In Experiment 2, IPOA achieved the best average flight distance, and it ranked first among all algorithms in the average convergence rate and average turning angle. For the average distance from obstacles, IPOA and MPOA both ranked second, demonstrating similarly strong performances. In Experiment 3, IPOA achieved the best average flight distance and average turning angle. Its average convergence rate was second, just behind HHO. However, HHO’s early convergence resulted in a poorer path quality. Regarding the average distance from obstacles, IPOA performed nearly on par with MPOA and POA. In Experiment 4, apart from having a slightly less favorable turning angle compared to POA, IPOA outperformed all other algorithms in every other metric. In Experiment 5, IPOA achieved the optimal results in terms of average flight distance and average iteration count, while its performance in the average turning angle and average distance to obstacles was relatively average. Conversely, IPOA’s performance in the average turning angle was not as strong.

Overall, across 20 metrics in the five experiments, IPOA achieved the best results in 13. Compared to POA, IPOA improved in path length, algorithm iterations, turning angle, and distance from obstacles by 8.39%, 14.21%, 3.34%, and 3.40%, respectively. Compared to MPOA, the improvements were similarly 5.51%, 3.92%, 1.49%, and 0.66%. Notably, since this set of experiments places significant emphasis on the path length cost, the clear improvement in path length achieved by IPOA strongly indicates that the algorithm’s optimization is successful.

### 6.4. Experiment on the Number of Different Obstacles

**Figure 10:**
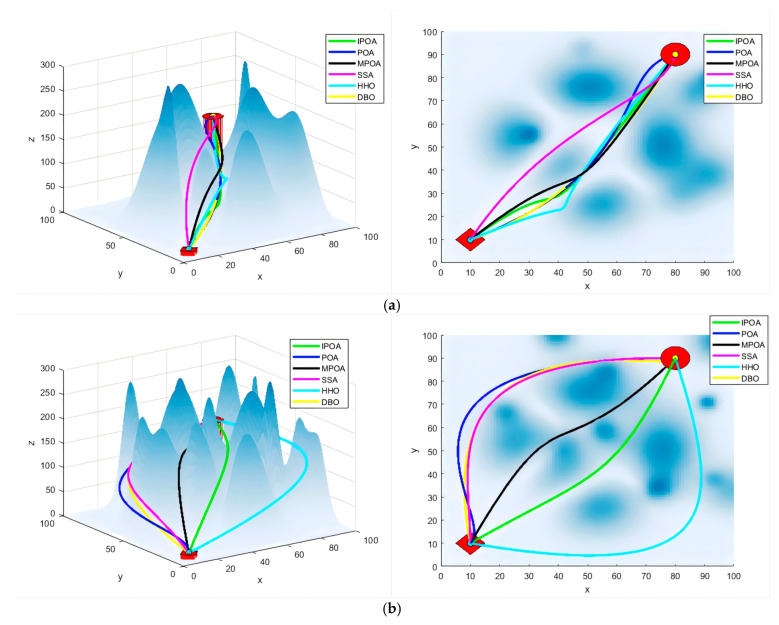
Flight simulation experiment of random maps (obstacles = 6 and 10). (**a**) Experiment 1 (obstacles = 6); (**b**) Experiment 2 (obstacles = 10).

The density of buildings in urban areas varies across different regions. To further evaluate IPOA’s path planning capabilities in complex environments, we conducted experiments on maps with obstacle counts of 6 and 10. Figure 10 illustrates the flight paths generated by each algorithm, showing both front views (left) and top views (right). To provide a comprehensive assessment of each algorithm’s performance, Table 7 presents a comparison of the evaluation metrics.

When the number of obstacles was six, compared to POA, IPOA improved the path length, algorithm iterations, turning angle, and distance to obstacles by 6.66%, 11.25%, 6.21%, and 3.22%, respectively. Compared to MPOA, the improvements were 3.69%, 7.28%, 0.22%, and 7.36%, respectively. When the number of obstacles was 10, compared to POA, IPOA improved the path length, algorithm iterations, turning angle, and distance to obstacles by 8.53%, 15.42%, 1.96%, and 2.58%, respectively. Compared to MPOA, the improvements were 5.57%, 8.41%, 0.39%, and 3.04%, respectively. Combined with the comparison data for eight obstacles in Section 6.3, it is evident that, as the number of obstacles increases, meaning the environment becomes more complex, the optimization efficiency of IPOA becomes more apparent in terms of path length and algorithm iterations. Overall, IPOA demonstrates excellent performance across various scenarios.

### 6.5. Simulation Experiment of Real Terrain

To evaluate the effectiveness of the algorithm in real-world environments, the RflySim simulation platform was used for UAV path planning experiments, simulating high-fidelity 3D modeling of real terrain. The RflySim platform, proposed by the Reliable Flight Control Group of Beihang University in 2021 [44], is an integrated simulation and development platform for UAV systems. RflySim adopts a model-based design approach and can be used for control and safety testing of unmanned systems. Quantitative analysis of the test platform’s results compared to actual experimental systems shows a high matching accuracy of over 90% (with 60% as the minimum required accuracy and 100% as a perfect match). Therefore, this simulation platform was chosen for the UAV application simulations in this study.

In the urban construction map, we modeled Dalian University as a 3D map. Dalian University is an interesting example, because it encompasses almost all elements of a modern city and has over ten thousand students and faculty members.

**Figure 11:**
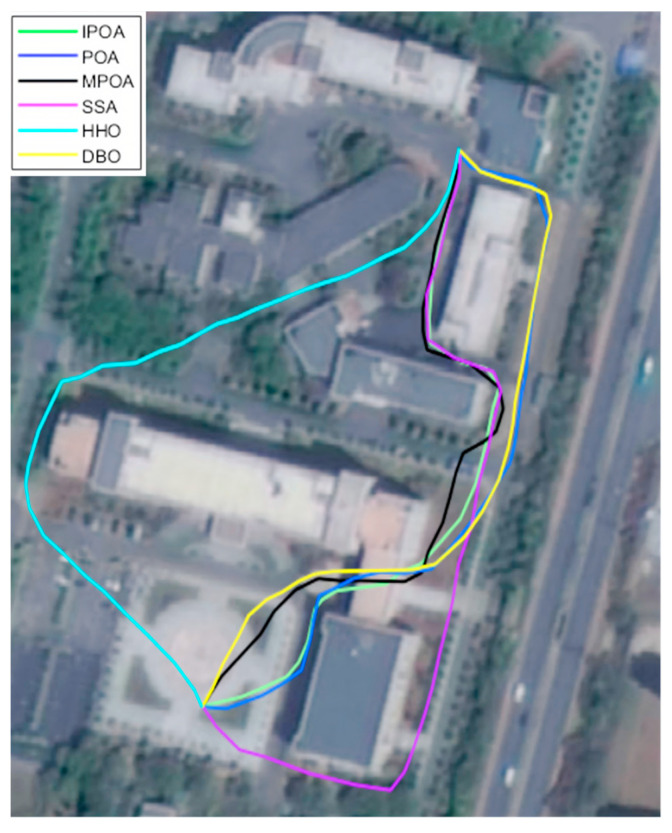
UAV flight trajectory at Dalian University.

On the RflySim platform, to obtain more accurate data, each algorithm was run 20 times. Using path length as the benchmark, Figure 11 displays the route for each algorithm. Using path length, turning angle, distance to obstacles, and flight time as the evaluation metrics, we recorded the data comparisons in Table 8.

It can be seen that IPOA achieves the shortest path length and the fastest flight time, as well as near-optimal turning angles and distances to obstacles. Compared to POA, the path length, turning angle, distance to obstacles, and flight time improved by 8.44%, 5.82%, 4.07%, and 9.36%, respectively. Similarly, compared to MPOA, the improvements were 4.09%, 0.76%, 1.85%, and 4.21%, respectively. This demonstrates that, in real-world scenarios, IPOA can provide a reliable solution for UAV path planning in urban environments.

### 6.6. Discussion

Through test functions and various map experiments, IPOA has demonstrated excellent performance. First, in the CEC2022 test functions, Wilcoxon rank-sum test, and box plot experiments, IPOA showed strong competitiveness compared to the other algorithms. This indicates that IPOA can handle a wide range of optimization problems. Second, in the path planning experiments under different conditions, the IPOA algorithm demonstrated both excellent performance and stability, enabling the creation of feasible and safe optimal trajectory paths for UAVs during mission execution. Lastly, when compared specifically to POA, it is clear that the multi-strategy approach proposed in this paper integrates and adapts well with POA.

Comparing IPOA to MPOA, it can be seen that their improvement directions are similar, which is why MPOA performs better than POA. In practical tests, IPOA outperforms MPOA, likely due to two key reasons: First, IPOA utilizes an iterative chaotic mapping method combined with ROBL during the population initialization phase, which enhances the population’s randomness and diversity. Additionally, compared to Cauchy mutation, the adaptive t-distribution exhibits stronger local search capabilities and can dynamically adjust the mutation step size based on the number of iterations, allowing for better balancing of global and local search abilities.

Although, in practical applications, most scenarios focus more on the algorithm’s impact on path length, in certain specific scenarios, other costs should carry greater weight (such as stricter obstacle avoidance requirements in fire rescue situations). Therefore, when the scenario changes, the cost functions and algorithm adjustments should be dynamic, which requires further experimental validation. Additionally, in complex environments, there is often multi-UAV collaboration. These aspects represent the limitations of this research. In future studies, we will explore more complex scenarios and multi-UAV path planning to make our research more applicable to real-world situations.

## 7. Conclusions

In the CEC2022 test suite, we studied the performance of IPOA in solving various optimization problems. The results showed that IPOA outperformed all other competing algorithms in 69.4% of the 36 metrics across three categories. This demonstrates that the improvement strategies proposed in this paper help enhance the performance of the original algorithm and expand its applicability.

In the UAV path planning experiments, we considered flight distance, turning angle, and collision threat, conducting experiments under various conditions. Given that efficiency is a key consideration for UAV task execution in real-world scenarios, this paper focused more on path length, conducting experiments on random maps and real city simulations. The results demonstrated that IPOA not only found shorter paths but also reduced turning angle consumption and enhanced safety to some extent, effectively meeting the optimization requirements we proposed. This proves the effectiveness of IPOA in practical applications.

Despite the significant achievements of IPOA in algorithm performance and path planning, this study also has some limitations. In urban environments, many special scenarios require continuous adjustment of the constraints in the cost function according to specific needs. For example, in fire rescue operations, UAVs should prioritize higher safety performance. When energy is limited, UAVs must reduce the number and magnitude of turns in order to conserve battery power. In future research, we will attempt to integrate hardware and develop adaptive intelligent optimization algorithms. Additionally, to improve task efficiency, we plan to explore information sharing and collaboration among multiple UAVs. To increase the practical value of this research, more advanced technologies like Unreal Engine 5 should be employed to create path planning scenarios for a broader range of cities.

## Figures and Tables

**Table 1:** CEC2022 test suite.

Function Type	No.	$F_{i}^{*}$	Functions
Unimodal function	$f_{1}$	300	Shifted and full Rotated Zakharov Function
Basic functions	$f_{2}$	400	Shifted and full Rotated Rosenbrock Function
	$f_{3}$	600	Shifted and full Rotated Expanded Schaffer Function
	$f_{4}$	800	Shifted and full Rotated Non-Continuous Rastrigin Function
	$f_{5}$	900	Shifted and full Rotated Levy Function
Hybrid functions	$f_{6}$	1800	Hybrid Function (N = 3)
	$f_{7}$	2000	Hybrid Function (N = 6)
	$f_{8}$	2200	Hybrid Function (N = 5)
Composition functions	$f_{9}$	2300	Composition Function (N = 5)
	$f_{10}$	2400	Composition Function (N = 4)
	$f_{11}$	2600	Composition Function (N = 5)
	$f_{12}$	2700	Composition Function (N = 6)
			Search range: ${\left[-100,\;100\right]}^{D}$

**Table 2:** Algorithm key parameter settings.

Algorithm	Parameters	Values
DBO	Deflection coefficient k	0.1
	Ball-Rolling dung beetles parameter b	0.1
	Stealing dung beetles parameter S	0.3
POA	Exploitation phase parameter R	0.2
HHO	Initial energy $E_{0}$	$\left[-1,1\right]$
	Jump strength J	(0, 2)
SSA	Safety threshold ST	0.6
	Proportion of finders PD	0.7
	Proportion of vigilante SD	0.2
MPOA	freedom parameter a	1
	Proportion of vigilante SD	0.2
IPOA	Exploitation phase parameter R	0.2
	Chaotic iterative mapping constant a	0.7
	Levy flight step parameters β	1.5

**Table 3:** Evaluation metrics for the CEC2022 test.

Functions	Metrics	IPOA	DBO [36]	POA [38]	SSA [34]	MPOA [43]	HHO [27]
$f_{1}$	Best	6989.62	13,293.43	14,564.86	17,967.23	10,699.83	19,435.23
	Mean	8676.68	16,612.76	17,567.55	21,293.43	12,879.42	24,783.45
	SD	544.85	1649.18	1293.82	1502.73	975.75	2182.99
	Ranking	1 1 1	3 3 5	4 4 3	5 5 4	2 2 2	6 6 6
$f_{2}$	Best	479.21	582.19	612.08	771.05	449.08	762.19
	Mean	522.86	673.45	698.46	820.58	489.18	872.11
	SD	33.19	66.20	55.74	34.94	31.89	65.95
	Ranking	2 2 2	3 3 6	4 4 4	6 5 3	1 1 1	5 6 5
$f_{3}$	Best	622.53	625.79	624.24	635.64	618.47	641.46
	Mean	625.98	634.85	635.43	646.26	626.66	658.25
	SD	3.99	6.15	7.61	9.25	7.83	13.26
	Ranking	2 1 1	4 3 2	3 4 3	5 5 5	1 2 4	6 6 6
$f_{4}$	Best	806.95	813.71	812.19	815.09	811.32	828.73
	Mean	822.56	825.58	821.91	825.28	823.06	844.89
	SD	5.28	9.96	6.47	4.22	6.04	9.08
	Ranking	1 2 3	4 5 6	3 1 4	5 4 1	2 3 2	6 6 5
$f_{5}$	Best	1943.50	2498.45	2559.36	3285.23	2008.52	2946.31
	Mean	2159.24	2954.46	2856.12	3804.16	2287.56	3214.68
	SD	149.42	296.45	193.45	256.01	180.82	369.14
	Ranking	1 1 1	3 4 5	4 3 3	6 6 4	2 2 2	5 5 6
$f_{6}$	Best	3347.46	8757.56	14,549.54	12,755.58	5489.24	16,763.65
	Mean	8480.64	54,207.68	66,946.34	107,686.45	12,657.65	226,784.77
	SD	2365.18	42,558.67	60,092.98	91,572.16	5469.12	181,273.25
	Ranking	1 1 1	3 3 3	5 4 4	4 5 5	2 2 2	6 6 6
$f_{7}$	Best	2009.65	2063.38	2088.24	2099.43	2018.23	2158.98
	Mean	2028.55	2148.33	2122.09	2176.61	2034.01	2176.38
	SD	13.95	67.465	28.35	52.07	18.45	14.75
	Ranking	1 1 1	3 4 6	4 3 4	5 6 5	2 2 3	6 5 2
$f_{8}$	Best	2207.08	2215.71	2219.55	2225.23	2204.25	2233.16
	Mean	2224.47	2231.36	2230.13	2237.66	2218.96	2266.65
	SD	9.17	15.18	8.89	10.28	8.82	29.19
	Ranking	2 2 3	3 4 5	4 3 2	5 5 4	1 1 1	6 6 6
$f_{9}$	Best	2526.98	2544.43	2533.38	2535.47	2539.18	2543.34
	Mean	2531.12	2591.28	2552.49	2588.45	2542.10	2579.63
	SD	2.29	28.17	18.12	38.95	0.91	19.26
	Ranking	1 1 2	6 6 5	3 3 3	3 5 6	4 2 1	5 4 4
$f_{10}$	Best	2501.16	2813.10	3321.58	3049.19	2535.88	4216.39
	Mean	2862.44	3219.37	4044.54	4615.24	2912.13	5891.29
	SD	203.64	285.56	363.79	625.50	212.98	598.75
	Ranking	1 1 1	3 3 3	5 4 4	4 5 6	2 2 2	6 6 5
$f_{11}$	Best	2942.12	2912.46	3687.47	3572.67	3557.19	3524.56
	Mean	3118.91	3342.65	3916.18	4041.52	3898.96	3619.74
	SD	109.50	252.14	189.67	244.29	177.06	178.11
	Ranking	2 1 1	1 2 6	6 5 4	5 6 5	4 4 2	3 3 3
$f_{12}$	Best	2938.44	2999.95	3010.74	2946.78	2994.06	3012.13
	Mean	2947.18	3007.47	3029.25	2957.64	3009.08	3028.86
	SD	5.22	6.64	10.19	12.38	10.42	18.93
	Ranking	1 1 1	4 3 2	5 6 3	2 2 5	3 4 4	6 5 6

**Table 4:** Wilcoxon rank-sum test results.

Function	DBO [36]	POA [38]	SSA [34]	HHO [27]	MPOA [43]
$f_{1}$	1.4643 × 10^−10^	3.0810 × 10^−08^	3.0198 × 10^−11^	3.0198 × 10^−11^	6.8462 × 10^−06^
$f_{2}$	0.0415	0.0571	0.0013	0.0002	0.6683
$f_{3}$	0.0013	1.5422 × 10^−07^	1.6836 × 10^−08^	1.4478 × 10^−07^	4.9364 × 10^−04^
$f_{4}$	0.0061	0.0324	0.0463	6.9641 × 10^−11^	0.0489
$f_{5}$	0.3415	0.2186	0.5003	0.4801	0.7390
$f_{6}$	6.4213 × 10^−08^	0.0004	9.9851 × 10^−04^	2.2891 × 10^−10^	3.8936 × 10^−05^
$f_{7}$	3.1019 × 10^−07^	0.0398	2.1341 × 10^−07^	3.7621 × 10^−09^	0.0466
$f_{8}$	4.7173 × 10^−05^	0.3571	3.3146 × 10^−08^	6.2251 × 10^−10^	0.8701
$f_{9}$	3.3640 × 10^−11^	3.0815 × 10^−09^	4.7093 × 10^−10^	3.1467 × 10^−11^	4.2914 × 10^−08^
$f_{10}$	0.5083	0.6412	0.6721	1.9851 × 10^−07^	0.8827
$f_{11}$	1.2375 × 10^−06^	0.1739	0.0391	5.4226 × 10^−09^	0.2634
$f_{12}$	0.0143	0.0188	0.0336	0.0002	0.0296

**Table 5:** Performance of the algorithm under different weight combinations.

	Weight Combination	Metrics	IPOA	DBO	HHO	POA	MPOA	SSA
Experiment 1	$\omega_{1}=0.7$, $\omega_{2}=0.15$, $\omega_{3}$ = 0.15	Best	33.04	43.96	102.17	45.59	37.64	79.35
		Mean	36.78	50.39	121.62	52.61	41.33	97.91
		SD	2.99	3.98	16.72	4.63	3.48	14.82
Experiment 2	$\omega_{1}=0.15$, $\omega_{2}=0.7$, $\omega_{3}$ = 0.15	Best	36.10	47.17	116.55	43.39	38.26	76.94
		Mean	41.84	55.32	132.74	52.17	43.37	92.38
		SD	4.15	6.24	15.57	6.61	3.96	16.67
Experiment 3	$\omega_{1}=0.15$, $\omega_{2}=0.15$,${\;\omega }_{3}$ = 0.7	Best	38.41	41.09	106.65	43.66	35.02	75.28
		Mean	45.25	58.63	133.87	55.81	46.77	97.96
		SD	5.71	17.25	15.07	6.80	6.42	14.06

**Table 6:** Evaluation metrics for the random map flight experiment (obstacles = 8).

Experiment	Metrics	IPOA	DBO	HHO	POA	MPOA	SSA
Experiment 1	Distance	219.67	233.54	241.56	246.41	234.43	238.88
	Iterations	72.1	79.0	89.1	87.2	70.4	77.5
	Turn/°	52.9	43.1	44.1	51.8	48.1	48.8
	Dis-obs	46.1	56.4	52.0	50.9	48.4	54.5
Experiment 2	Distance	180.39	199.24	222.45	204.76	193.63	208.65
	Iterations	60.6	69.3	77.2	73.5	66.9	80.1
	Turn/°	44.2	48.6	58.7	50.4	45.8	47.4
	Dis-obs	49.3	51.2	42.4	47.6	49.3	58.7
Experiment 3	Distance	187.66	200.61	208.30	201.97	195.24	200.03
	Iterations	78.7	82.6	61.1	79.2	80.8	89.4
	Turn/°	49.9	56.7	77.1	57.2	53.1	58.1
	Dis-obs	44.7	43.1	41.3	43.6	42.8	43.5
Experiment 4	Distance	189.87	202.62	204.93	200.94	202.65	203.56
	Iterations	86.1	95.3	99.4	101.1	90.2	95.7
	Turn/°	46.2	46.9	61.0	43.3	49.4	53.8
	Dis-obs	59.7	65.2	64.8	66.9	64.1	66.4
Experiment 5	Distance	183.46	194.73	200.65	196.48	191.63	192.42
	Iterations	62.3	73.2	66.4	71.4	65.9	70.5
	Turn/°	54.3	52.1	67.6	54.6	55.1	50.4
	Dis-obs	42.9	43.8	48.6	44.2	41.3	46.5

**Table 7:** Evaluation metrics for the random map flight experiment (obstacles = 6 and 10).

Number of Obstacles	Metrics	IPOA	DBO	HHO	POA	MPOA	SSA
6	Distance	184.81	192.14	198.74	198.01	191.64	199.42
	Iterations	53.0	64.3	67.9	63.1	60.4	72.5
	Turn/°	45.3	41.6	42.1	48.3	45.4	43.8
	Dis-obs	54.1	62.4	61.2	55.9	58.4	60.5
10	Distance	190.96	205.37	218.33	208.79	202.24	210.23
	Iterations	72.9	86.7	101.1	86.2	79.6	88.1
	Turn/°	49.9	50.7	47.1	50.9	50.1	54.6
	Dis-obs	41.4	43.4	41.7	42.5	42.7	45.6

**Table 8:** Evaluation metrics for the simulation experiment of real terrain.

Metrics	IPOA	DBO	HHO	POA	MPOA	SSA
Distance (m)	286.74	318.88	342.66	313.19	298.97	322.81
Turn/°	51.7	55.6	51.5	54.9	52.1	53.8
Dis-obs (m)	2.11	2.04	2.13	2.24	2.16	2.42
Time (s)	20.23	22.77	24.16	22.32	21.12	22.91

## Data Availability

The raw data supporting the conclusions of this article will be made available by the authors on request.

## Appendix A

**Table A1:** Summary of the notations in IPOA.

Module Name	Notation	Meaning of Notation
POA	*N*	the number of population members
	*m*	the dimensionality counter of the problem variables
	*D*	the dimensionality of the problem variables
	$l_{i}$	the lower bounds of the problem variable
	$u_{j}$	the upper bounds of the problem variable
	*I*	random integer that can be either 1 or 2
	*t*	the iteration counter
	*T*	the maximum number of iterations
	*R*	a parameter that can be set between 0 and 1
Iterative chaotic mapping	*a*	control parameter
ROBL	*k*	refraction scale factor
	*n*	index of refraction
Nonlinear inertia weight factor	ω	nonlinear inertia weight factor
Levy flight	β	parameter controlling step size
	*s*	the Levy flight path factor
Adaptive t-distribution variation	*v*	degrees of freedom

**Table A2:** Summary of the notations in the evaluation metrics.

Evaluation Metrics	Meaning of Notation
Best	closest to the global optimum
Mean	the average of all results
SD	the degree of dispersion of the results
Distance	the average path length
Iterations	the average number of iterations of the algorithm
Turn/°	the average deflection angle difference between two adjacent nodes on the path
Dis-obs	the average distance between the current node and the nearest obstacle
Time	the average flight time
